# The Role of Prokineticin 2 in Oxidative Stress and in Neuropathological Processes

**DOI:** 10.3389/fphar.2021.640441

**Published:** 2021-03-01

**Authors:** Roberta Lattanzi, Cinzia Severini, Daniela Maftei, Luciano Saso, Aldo Badiani

**Affiliations:** ^1^ Department of Physiology and Pharmacology “Vittorio Erspamer”, Sapienza University of Rome, Rome, Italy; ^2^ Institute of Biochemistry and Cell Biology, IBBC, CNR, Rome, Italy

**Keywords:** neurotoxicity, neurodegenerative diseases, prokineticin receptors, Prokineticin receptor antagonists, prokineticin 2

## Abstract

The prokineticin (PK) family, prokineticin 1 and Bv8/prokineticin 2 (PROK2), initially discovered as regulators of gastrointestinal motility, interacts with two G protein-coupled receptors, PKR1 and PKR2, regulating important biological functions such as circadian rhythms, metabolism, angiogenesis, neurogenesis, muscle contractility, hematopoiesis, immune response, reproduction and pain perception. PROK2 and PK receptors, in particular PKR2, are widespread distributed in the central nervous system, in both neurons and glial cells. The PROK2 expression levels can be increased by a series of pathological insults, such as hypoxia, reactive oxygen species, beta amyloid and excitotoxic glutamate. This suggests that the PK system, participating in different cellular processes that cause neuronal death, can be a key mediator in neurological/neurodegenerative diseases. While many PROK2/PKRs effects in physiological processes have been documented, their role in neuropathological conditions is not fully clarified, since PROK2 can have a double function in the mechanisms underlying to neurodegeneration or neuroprotection. Here, we briefly outline the latest findings on the modulation of PROK2 and its cognate receptors following different pathological insults, providing information about their opposite neurotoxic and neuroprotective role in different pathological conditions.

## Introduction

Prokineticins (PKs) represent a family of chemokine-like peptides characterized by their ability to induce hyperalgesia and gastrointestinal motility. The first PK was discovered about twenty years ago and named Bv8 to denote its origin, the skin secretions of *Bombina variegata,* and its molecular weight, 8 kDa (Mollay et al., 1999). The family also includes MIT-1 (Mamba Intestinal Toxin 1) isolated from the venom of Black Mamba (Schweitz et al., 1999) and the mammalian Prokineticin 1 (PROK1) and Prokineticin 2 (PROK2) (Li et al., 2001).

PKs structure consists of a highly conserved hexapeptide in N-terminal position and ten cysteine residues, that form five disulphide bridges, giving to the molecule a very compact structure (Negri and Ferrara, 2018). This represents a substantial difference compared to that of chemokines characterized, instead, by four to six cysteine residues.

PKs effects are mediated by biding with two G protein-coupled membrane receptors: prokineticin receptor 1 (PKR1) and 2 (PKR2) which share an 85% identity in the amino acid sequence (Lin et al., 2002; Soga et al., 2002; Negri et al., 2009).

PKs and PKRs are present in a multiplicity of organs and tissues, including the central nervous system (CNS), gonads, adrenal gland, placenta, uterus, gastro-intestinal tract, heart, bone marrow and blood. Not surprisingly, the PKs have been shown to participate in a bewildering variety of physiological actions comprehending the coordination of complex behaviors as circadian rhythms, muscle contractility, reproduction, angiogenesis, neurogenesis, haematopoiesis, immune responses and pain perception (Negri and Ferrara, 2018).

Of particular relevance to the topic of this review, is the extended distribution of PKs and their receptors in the mammalian organs and tissues. In CNS the ligands are less expressed than the receptors. PROK1 is mainly present in the tractus solitarius and in the cerebellum. PROK2 is evident in several forebrain regions, mainly in the hypothalamus and in the suprachiasmatic nucleus (Cheng et al., 2002; Cheng et al., 2006), and in the spinal cord (Maftei et al., 2014). Regarding the receptors, their relative density in the brain changes as a function of discrete areas, but in most regions PKR2 is expressed more than PKR1 (Cheng et al., 2006; Negri and Ferrara, 2018). From a neurodevelopmental point of view, both PKR1 and PKR2 can be detected as early as at embryonic day 7 in mouse and in rat (Negri et al., 2007). In rat embryos, both PKR1 and PKR2 are highly expressed in the neuroepithelium lining the ventricles, olfactory bulb, Gasser-ganglion and Dorsal Root Ganglia (DRG). However, in neonatal and adult rat brain only PKR2 is still expressed at high levels in multiple areas, whereas PKR1 is detected at low density only in the cortex (Cheng et al., 2006; Negri et al., 2007; Zhang et al., 2009). PKR1 and particularly PKR2 are also present in the dorsal horns of the spinal cord and in DRG, indicating their role in the transmission of noxious stimuli (Maftei et al., 2014; Negri and Maftei, 2018).

The distribution of PROK2 and PKRs in the CNS varies as a function of cellular types. Studies conducted in primary cultures of neurons, astrocytes and microglia, obtained from embryo or neonate brain mice, have shown that PKR2 is mostly expressed in neurons, whereas PROK2 and PKR1 are mainly expressed in astrocytes and microglia (Koyama et al., 2006; Severini et al., 2015).

The widespread distribution of PROK2 and its receptors, mainly PKR2, in the CNS underlies not only their role in physiological functions, but also their participation in neuropathological processes. Indeed, PROK2 may be over-expressed following a series of pathological insults, such as hypoxia, reactive oxygen species (ROS), amyloid β (Aβ) and excitotoxic glutamate, suggesting its involvement in numerous processes that lead to neuronal death. Interestingly, in diverse neuronal populations and in different pathological conditions PROK2 can show both neurotoxic and neuroprotective effects **(** Table 1 **).** We will focus on the response of the PK system to a variety of pathological insults and on the mechanisms implicated in its neurotoxic and neuroprotective effects.

**TABLE 1:** PK system modulation related to neurotoxic or neuroprotective activity following different pathological insults.

Pathological insult	PROK2 activity	Experimental model	Results
*Oxidative stress*	Neurotoxic	-Primary cortical cell cultures	-Hypoxia and ROS: ↑ PROK2 mRNA mainly in neurons (Cheng et al., 2012)
		-*in vivo* stroke models	-Ischemic cortex and striatum: ↑ PROK2 mRNA (Cheng et al., 2012) -PROK2 i.c.v. injection after stroke: ↑ infarct volume (Cheng et al., 2012) - PKRs antagonist: ↓ infarct volume, ↓ central inflammation and improves behavioral outcome (Cheng et al., 2012)
	Neuroprotective	-*in vitro* model of focal cerebral ischemia	-Bv8: ↓ the OGD-induced necrotic neuronal death in cortical cultures and hippocampal slices activating ERK, Akt and GSK3-β pathways (Landucci et al., 2016) -PKRs antagonist reverts the Bv8 effects (Landucci et al., 2016)
		*-in vitro* model of ischemic tolerance (NMDA preconditioning)	-Hippocampal slices: ↑ PROK2, PKR1 and PKR2 mRNA and protein (Landucci et al., 2016) -PKRs antagonist: ↓ PROK2 protein in hippocampal slices (Landucci et al., 2016)
		-H9c2 cardiomyocytes cell line from rat heart	-PROK2 treatment or PKR1 overexpression: ↓ oxidative stress-induced apoptotic cell death activating the Akt/mTOR and AKT/GSK3β pathways (Urayama et al., 2007; Su et al., 2020; Yang et al., 2020)
*Beta amyloid*	Neurotoxic	-Primary cortical cell cultures	-Aβ_1-42_: ↑ PROK2, PKR1 and PKR2 mRNA and protein levels in neurons and astrocytes (Severini et al., 2015) -Bv8, at pM concentrations, ↑ neuronal apoptosis (Severini et al., 2015) -PKRs antagonist: ↓ Aβ- and Bv8-induced neuronal apoptosis (Severini et al., 2015) and ↓ kainate-evoked current increase Aβ- induced (Caioli et al., 2017)
		-Tg2576 transgenic mouse model of AD↓	-Cortex and hippocampus: ↑ PROK2 mRNA (Lattanzi et al., 2019) -PKRs antagonist: ↓ LTP impairment in hippocampal slices (Severini et al., 2015)
		-*in vivo* non-transgenic rat model of AD	-Aβ_1-42_ i.c.v. injection: ↑ PROK2, PKR1 and PKR2 mRNA and protein in prefrontal cortex and hippocampus (Severini et al., 2015; Lattanzi et al., 2019; Maftei et al., 2019) -PKRs antagonist: ↓ PROK2 levels, ↓ glial activation and neuronal death, restores the neurogenesis in DG and ↓ the Aβ-induced memory deficits (Maftei et al., 2019)
		-AD patients	-↑ PROK2 mRNA in hippocampus and ↑ PROK2 protein in serum (Lattanzi et al., 2019)
*Glutamate*	Neurotoxic	-Primary cortical cell cultures	-NMDA and excitotoxic glutamate: ↑ PROK2 mRNA mainly in neurons (Cheng et al., 2012) -Bv8, at pM concentrations, ↑ AMPA currents (Caioli et al., 2017) -PKRs antagonist protects neurons from kainate-induced death (Caioli et al., 2017)
		-Primary striatal cell cultures	-Excitotoxic glutamate: ↑ PROK2 mRNA (Cheng et al., 2012)
	Neuroprotective	-Primary cortical cell cultures	-Bv8, at μM concentrations protects the cortical neurons against NMDA toxicity (Melchiorri et al., 2001)
		-Primary mouse astrocytes	-PROK2 and IS20 (a PKR1 agonist): ↑ astrocytes uptake of extracellular glutamate (Neal et al., 2018)
		-*in vitro* model of ischemic tolerance	-NMDA-induced OGD tolerance: ↑ PROK2/PKRs protein (Landucci et al., 2016) -PKRs antagonist: ↓ PROK2 and ↓ the development of NMDA-induced OGD tolerance and blocks the NMDA-induced activation of ERK1/2 and Akt (Landucci et al., 2016)
*Dopaminergic neurons toxicity*	Neuroprotective	-N27 dopaminergic neuronal cell cultures	-MPP^+^ and TNFα: ↑ PROK2 mRNA and protein in DAergic neurons (Gordon et al., 2016) -PROK2: Protects against MPP^+^-mediated oxidative stress, mitochondrial dysfunction and neuronal cell death activating ERK and Akt pathways (Gordon et al., 2016)
		-Primary mesencephalic neuronal cell cultures	-PROK2: ↓ MPP^+^-induced DAergic neuronal loss (Gordon et al., 2016)
		-*in vivo* MPTP model of PD	-DAergic neurons of nigral tissue: ↑ PROK2 protein (Gordon et al., 2016) -PROK2: ↓ MPTP-induced behavioral deficits and DAergic degeneration (Gordon et al., 2016) -PKRs antagonist: ↑ DAergic degeneration and **↑** the behavioral deficits (Gordon et al., 2016) -IS20: Protects against MPTP-induced reduction of astrocytic A2 antiinflammatory phenotype and ↓ expression of proinflammatory factors (Neal et al., 2018)
		-MitoPark mice transgenic model of PD	-Nigral DAergic neurons: ↑ PROK2 following both acute and progressive DAergic degeneration (Gordon et al., 2016)
		-PD patients	-Nigral DAergic neurons: ↑ PROK2 protein (Gordon et al., 2016)

## PKs Involvement in the Response to Pathological Insults

### Oxidative Stress

Oxidative stress indicates that cells suffer the harmful effects induced by excessive levels of ROS, potentially leading to damage to lipids, proteins or nucleic acids. Oxidative DNA damage occurs immediately after ischemic stroke and is generally reversible (Cui et al., 2000; Lan et al., 2003). This last point appears particularly interesting because it offers the possibility of a therapeutic treatment.

It has been reported (Cheng et al., 2012) that PROK2 mRNA levels increase *in vitro,* in primary cortical cultures following hypoxia and ROS treatment, and also in an *in vivo* model of stroke obtained by transient intraluminal middle cerebral artery occlusion. The PROK2 increase was observed in the ischemic cortex and striatum and was proportionally to the severity of damage, indicating that the stroke-induced PROK2 up-regulation is a consequence of the ischemic damage. The PROK2 overexpression proved to be harmful since the exogenous PROK2 post-stroke delivery worsened the ischemic injury, whereas blocking PROK2 expression reduced the infarct volume and central inflammation and improved behavioral outcome, suggesting that impairing the PROK2 levels could be therapeutic The harmful effect of PROK2 is also supported by the signal transduction pathways activation. Indeed, treatment of primary neuron cultures with PROK2, after exposure to glutamate excitotoxicity or oxygen glucose deprivation (OGD), increased both pERK1,2 and pSAP/JNK MAPK levels without changes in pAkt levels, indicating that these pathways are partially responsible for PROK2-induced deleterious effects in ischemic injury. Although ERK1,2 and Akt signaling exert mostly beneficial effects (Zhao et al., 2005; Zhao et al., 2006), they also may be involved in deleterious effects, increasing ischemic damage (Sawe et al., 2008). On the whole, the authors suggested that PROK2 is an endangering mediator for ischemic brain injury and a compelling target for stroke treatment.

Conversely, a neuroprotective role for PROK2 has been also reported in cerebral ischemia (Landucci et al., 2016). In this study, Bv8 (the amphibian PROK2 homologue) exerted neuroprotective effect in two *in vitro* models of cerebral ischemia, cortical cell cultures and in organotypic hippocampal slices exposed to OGD. Furthermore, the ischemic tolerance, induced by the exposure of hippocampal slices to a preconditioning sub-toxic N-methyl-d-aspartate (NMDA) stimulus before the exposure to OGD (Gerace et al., 2012), led to an increase of PROK2/PKRs mRNA and protein levels which was inhibited by PC7, a PKRs antagonist. The PROK2 neuroprotective effect was associated to the activation of the ERK1/2 and Akt transduction pathways (Landucci et al., 2016).

A possible explanation for the discrepancy between neuroprotective and neurotoxic effects exerted by PROK2, *in vitro*, could be ascribed to the different range of concentrations used. Protective/pro-survival effect of PROK2 was generally obtained with nanomolar concentrations of Bv8/PROK2, able to activate the pro-survival ERK1/2 and Akt transduction pathways (Melchiorri et al., 2001; Negri et al., 2005; Ng et al., 2005; Urayama et al., 2008; Gasser et al., 2015). Conversely, the pro-apoptotic effect of PROK2 was obtained at picomolar concentrations, acting on the SAP/JNK pathway under excitotoxic or OGD conditions (Cheng et al., 2012; Gasser et al., 2015; Severini et al., 2015).

Protective effects of PROK2 were also demonstrated in other organ systems. PROK2, through PKR1 binding and PKR1 overexpression, protected the cardiomyocytes from oxidative stress-induced apoptosis through Akt pathway activation. These effects were completely nullified by siRNA-PKR1. In the same work, in an *in vivo* animal model of myocardial infarction, induced by coronary ligation*,* the authors demonstrated that PKR1 gene therapy preserved the myocardial function by blocking the apoptosis and stimulating angiogenesis (Urayama et al., 2007; Urayama et al., 2008). In line with this data, recent studies have confirmed that PROK2 exerts its protective role on cardiomyocytes by activating the Akt/mTOR (Su et al., 2020) and AKT/GSK3β (Yang et al., 2020) signaling pathways.

### Aβ Toxicity

The presence of extracellular Aβ plaques, tau neurofibrillary tangles and gliosis are associated with the pathogenesis of Alzheimer’s disease (AD) (Akiyama et al., 2000; Crews and Masliah, 2010). Activated astrocytes and microglia are the main source of pro-inflammatory factors: among them, chemokines and chemokine receptors have been shown to be over-expressed in AD brain (Rubio-Perez and Morillas-Ruiz, 2012; Zuena et al., 2019). The new chemokine-like peptide PROK2 and PKRs were found to be modulated in response to Aβ insult. Indeed, in mixed primary rat cortical cultures and in cortex and hippocampus from Aβ-treated rats, PROK2, PKR1, and PKR2 mRNA and protein levels were significantly up-regulated by Aβ treatment (Severini et al., 2015; Caioli et al., 2017). PROK2 was significantly increased in astrocytes, the principal cells producing chemokines after Aβ insult (Johnstone et al., 1999; Severini et al., 2013) and in neurons; PKR1 was mainly increased in neurons while PKR2 in both neurons and astrocytes (Severini et al., 2015).

**FIGURE 1:**
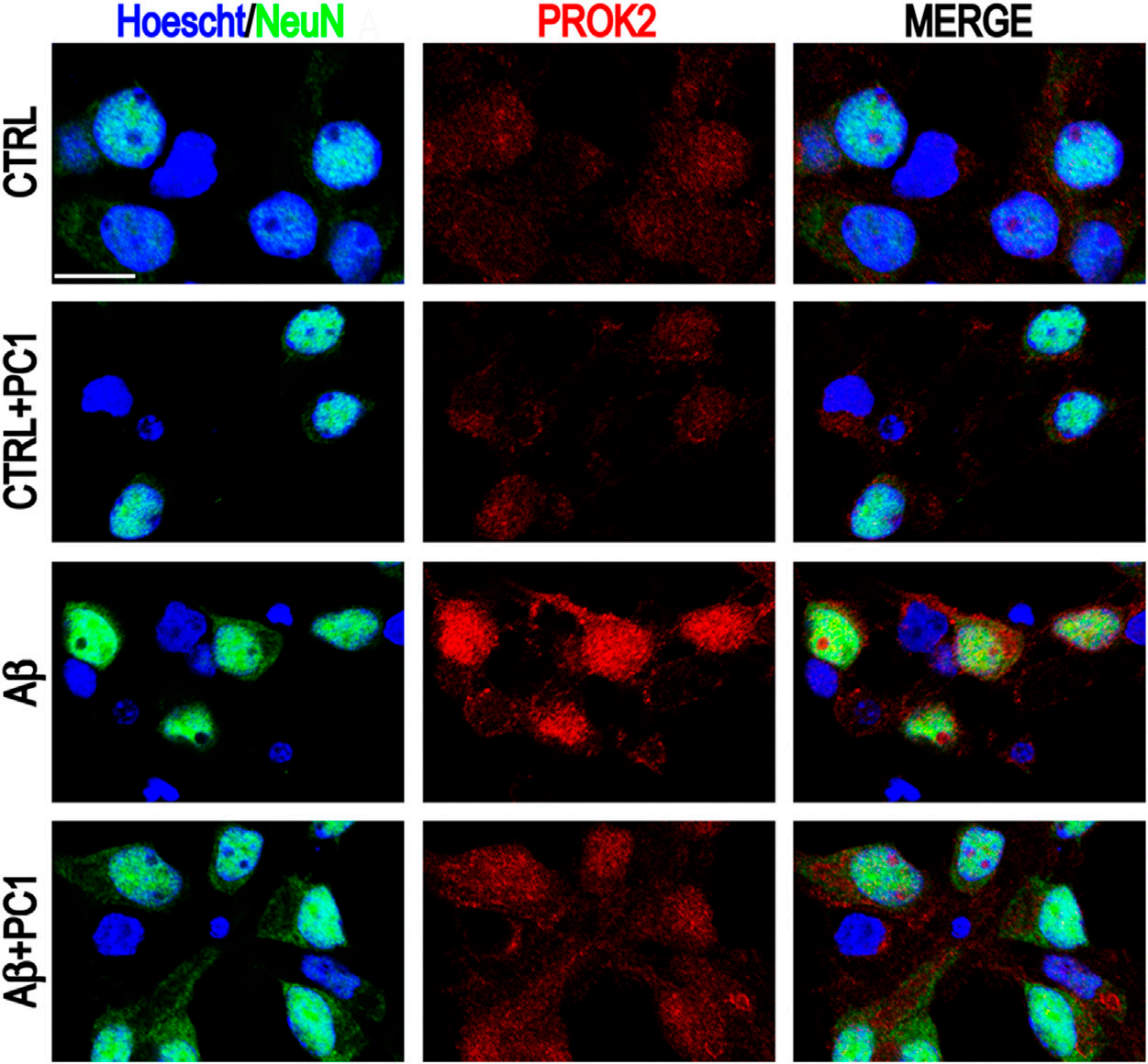
Representative immunofluorescence images of cultured mixed cortical neurons (CNs) stained with anti-PROK2 antibody (red) in control conditions (CTRL) and after 48 h of Aβ_1–42_ treatment alone or in the presence of PC1 (200 nM). Neurons were stained with NeuN (green), and nuclei with Hoecsht (blue). Scale bar: 15 μm (Severini et al., 2015).

The neurotoxic/deleterious activity of the PK system in Aβ-induced neurotoxicity was further demonstrated by the ability of PC1, a PKRs antagonist (Balboni et al., 2008; Congiu et al., 2014; Lattanzi et al., 2014), to concentration-dependently prevent Aβ-induced apoptosis and to reduce the PROK2 increase**.** Indeed, the exposure of primary cortical cultures to Aβ_1-42_ strongly induced PROK2 overexpression in neurons that was significantly decreased by PC1 treatment **(** Figure 1 **),** impairing the pro-apoptotic signaling and confirming previous data which demonstrate the PC1 ability to reduce PROK2 expression and storage in mice spinal neurons and astrocytes following peripheral nerve injury (Maftei et al., 2014; Lattanzi et al., 2015).

The neurotoxic role of PROK2 in Aβ toxicity is supported also by the Bv8 ability to induce in cortical brain cultures, at picomolar concentrations, apoptosis comparable to that exerted by Aβ, pointing out that such a low concentrations could be in accordance with the small amount of PROK2 eventually released by the Aβ treatment. The Bv8-induced apoptosis was prevented by co-administration of PC1, which prevented also the long-term potentiation (LTP) impairment in hippocampal slices of Tg2576 transgenic mice compared with age-matched wild-type controls (Severini et al., 2015). The PC1 capability to antagonize Aβ toxicity and LTP impairment in the Tg2576 transgenic mice, indicates that damage-induced PROK2 overexpression is deleterious and that blocking the PK system may represent a therapeutic strategy.

In another study it was reported that, in neurons from primary cortical cultures, PC1 reverted the increase of ionic current induced by Aβ trough the AMPA (α-amino-3-hydroxy-5-methyl-4-isoxazole-propionic acid) receptors and protected the cortical neurons against kainite-induced death. Interestingly, still in cortical neurons, picomolar concentrations of Bv8 increased both evoked and spontaneous AMPA currents and this effect was nullified by PC1.

The Bv8 effect was not due to an increase of AMPA receptor subunit expression. Instead, it seems to be mediated by activation of the intracellular protein kinase C (PKC) since the PKC inhibitor Go6983 counteracted the effect (Caioli et al., 2017).

A recent paper highlights a neuroprotective role of PC1 on Aβ-induced memory impairment in rats through the modulation of the PK system. Aβ intracerebroventricular (i.c.v) infusion induced a strong cognitive dysfunction and a significant up-regulation of PROK2 and PKRs in cortical and hippocampal neurons and astrocytes. Subcutaneous treatment with PC1 prevented the Aβ-induced memory reduction restoring not only the balance of the whole PK system but also the physiological neurogenesis in Dentate Gyrus (Maftei et al., 2019) which results compromised in AD brains (Li et al., 2008). Moreover, a significant PROK2 up-regulation was also demonstrated in brain of Tg2576 transgenic AD mice and in both brain and serum of AD patients (Lattanzi et al., 2019).

The PK system seems to be a key mediator in the Aβ-induced neuronal damage and PK antagonists may represent new therapeutic drugs to ameliorate the AD progression. The significant PROK2 increase in AD patient’s serum could indicate PROK2 as a potential blood-based biomarker of the pathology.

### Glutamatergic Toxicity

A growing body of evidence suggests that glutamate receptor-mediating excitotoxicity plays a pivotal role in the pathogenesis of many neurodegenerative diseases such as AD, Huntington’s, Parkinson’s and Amyotrophic Lateral Sclerosis disease (Lipton and Rosemberg, 1994; Blandini et al., 1996).

The exposure of primary cortical cultures to excitotoxic glutamate and NMDA induced PROK2 mRNA overexpression. The glutamate-induced PROK2 up-regulation depended by NMDA receptor activation, required extracellular Ca^2+^ and was present mainly in neurons (Cheng et al., 2012). Furthermore Bv8 increased, in cortical neurons, the AMPA currents evoked by kainate perfusion and its effect was annulled by PC1 (Caioli et al., 2017), indicating that a dysregulation of the PK system balance may have a deleterious role in glutamatergic neurotoxicity.

However, the harmful activity of Bv8 in glutamate neurotoxicity disagrees with previously reported data which demonstrated that, in murine cortical cultures, Bv8 protected against NMDA-induced excitotoxicity through the activation of the MAPK and PI3-K pathways (Melchiorri et al., 2001). In support of these data, other recent studies reported a protective role of PROK2 in a model of ischemic tolerance *in vitro,* induced by the pre-exposure of hippocampal slices to a preconditioning subtoxic concentration of NMDA before the exposure to OGD. Indeed, the block of PKRs prevented the development of the NMDA-induced OGD tolerance and the subsequent PROK2 increase, and blocked the activation of the pro-survival ERK1/2 and PI-3 kinase/Akt signaling pathways normally promoted by the NMDA preconditioning stimulus (Landucci et al., 2016).

In another study, Neal and colleagues (Neal et al., 2018) suggested that the neuroprotective role of PROK2 may be due also to its ability to increase the uptake of extracellular glutamate by astrocytes through the upregulation of the glutamate transporter GLAST. Taken together, these data suggest that PROK2/PKRs signaling may play an important role also in molecular mechanisms underlying neuroprotection.

### Dopaminergic Neurons Toxicity

The degeneration and the progressive loss of the dopaminergic (DA) neurons in the substantia nigra pars compacta (SNpc) is considered the most important hallmark of Parkinson's disease (PD).

The involvement of PROK2 in dopaminergic neurodegeneration was proposed since its expression is strongly increased, both *in vitro* and *in vivo*, in DA neurons during the neurodegeneration process. *In vitro*, the exposure of the dopaminergic neurons to the TNFα (tumor necrosis factor alpha) or parkinsonian neurotoxin 1-methyl-4-phenylpyridinium (MPP^+^), important contributors to the pathogenesis of PD, increased the expression of both PROK2 mRNA and secreted protein in the early stage of neuronal death (Gordon et al., 2016). In agreement, *in vivo* data from preclinical mouse models of PD, MPTP model (Jackson-Lewis and Przedborski, 2007) and transgenic MitoPark mice (Ekstrand and Galter, 2009), indicated an early PROK2 upregulation in nigral DA neurons before the onset of neuronal degeneration and motor deficits. These data indicate that PROK2 is an inducible secreted mediator upregulated precociously in the DA neurons in response to neurotoxic stress. In addition, PROK2 protein expression was found elevated also in nigral tissue (Gordon et al., 2016) and in serum from PD patients (Schirinzi et al., 2021). PROK2 serum increase correlates with CSF Aβ increase and lactate decrease, suggesting that PROK2 may have a protective role at synaptic level and an antioxidant action promoting recovery from mitochondrial damage.

Regarding PKR2, its constitutive expression in SN remained unaltered in both MPTP-treated mice and in PD patients’ brain (Gordon et al., 2016).

PROK2 overexpression in DA neurons has a neuroprotective role since its administration, via selective interaction with the cognate PKR2, reduced the MPP^+^-induced neuronal cell death, oxidative stress and mitochondrial dysfunction and promoted the mitochondrial biogenesis in dopaminergic neurons. PROK2 exerted its protective effects through the activation of ERK and Akt signaling pathways, and the upregulation of PGC-1α and TFAM, the main mediators of mitochondrial biogenesis (Gordon et al., 2016). The protective effect of PROK2 could be also associated to its ability to induce an anti-inflammatory A2 phenotype in astrocytes. Indeed, in astrocytes cultures, thought selective interaction with the PKR1, PROK2 reduced the levels of ROS and pro-inflammatory factors, such as TNFα and IL1β, increased the astrocytic uptake of glutamate and the expression of neuroprotective factors such as the antioxidant arginase-1 or Nrf2 (Neal et al., 2018). Nrf2 overexpression in astrocytes, as well as the astrocytic ability to remove the extracellular glutamate, has been demonstrated to protect the dopaminergic neurons from death (Yao et al., 2005; Gan et al., 2012).

Moreover, PROK2 administration into the mouse striatum, significantly improved the MPTP-induced locomotor deficits and protected against dopaminergic neuronal degeneration, while the administration PKRA7, a PKRs antagonist, blocked the protective function of PROK2 (Gordon et al., 2016; Neal et al., 2018).

Since glial activation has been reported in many neurodegenerative disorders, including PD, and this activation contribute to the disease progression (Guzman-Martinez et al., 2019; Li et al., 2019), the upregulation of PROK2 in dopaminergic neurons represents not only a local protective mechanism against neuronal death but could also play an important role in neuron-glia cross-talk. Given the neuroprotective activity of PROK2, it is possible to speculate that agonists for PK receptors might represent a novel neuroprotective therapeutic approach that can slow down or halt the dopaminergic neuronal degeneration in PD.

## Conclusions

The data presented in this review bring evidence that activation of the PK system by various pathological stressors can exert both neuroprotective and neurotoxic effects in diverse experimental conditions and human pathologies. These studies highlight that PROK2 neuronal activity depends on various factors including the specific neuronal population, the amount of PROK2 released and the type of receptor activated. Depending on the amount released by pathological insults (in the range of pmol or nmol) PROK2 could activate a different fraction of PKR1 and PKR2 and different, sometimes opposite, transduction pathways. In the CNS, the proper role of PKR1 and PKR2 regarding the mechanisms leading to neuroprotection or neurodegeneration has not been fully understood yet, mainly for the absence of specific PK receptor subtype antagonists. The PROK2 pro-survival/neuroprotective effects at nanomolar concentrations range requires mainly the activation of ERK1/2 and Akt signaling pathways, whereas the damaging effects of PKs picomolar concentrations seems to involve ERK1,2 and SAP/JNK pathway, without involvement of Akt signaling pathways.

Thus, further studies are needed to clarify how the receptor-dependent signaling can modulate PROK2 responses by analyzing PKR1-or PKR2-deficient mice with different treatments that induce neuroprotection or neurodegeneration. Understanding the role of PROK2 and its receptors in neuronal death and/or survival may be a crucial step in clarifying the mechanisms involved in various neuropathologies.
